# Needle in the heart: a rare case of cardiac tamponade caused by a migrated foreign body and mimicking ST segment elevation myocardial infarction

**DOI:** 10.1186/s12872-021-01950-6

**Published:** 2021-03-17

**Authors:** Miklós Pólos, Dominika Domokos, Cristina-Maria Şulea, Kálmán Benke, Gergely Csikós, Andrea Nagy, Réka Skoda, András Szabó, Eperke Merkel, István Hartyánszky, Zoltán Szabolcs, Béla Merkely, Dávid Becker

**Affiliations:** 1 Heart and Vascular Center, Semmelweis University, Varosmajor Str. 68, 1122 Budapest, Hungary; 2 George Emil Palade University of Medicine, Pharmacy, Science, and Technology of Targu Mures, Targu Mures, Romania; 3 Department of Anesthesiology and Intensive Care, Semmelweis University, Budapest, Hungary

**Keywords:** Cardiac tamponade, Foreign body migration, STEMI, Case report

## Abstract

**Background:**

Pericardial tamponade is a serious condition which may eventually lead to severe haemodynamic disturbances and cardiac arrest. It is most often caused by the accumulation of fluid inside the pericardium, as a result of different aetiological factors such as pericarditis, neoplastic diseases, lymphatic dysfunctions, or idiopathic pericardial disease. Pericardial tamponade can develop after cardiac surgical procedures or as a complication of myocardial infarction. Collection of blood inside the pericardial sack can be the result of pericardial or cardiac trauma. It is exceedingly rare for the injury to be caused by a migrating foreign body. Although a typical picture of pericardial tamponade has been previously described, the disorder may clinically resemble an acute myocardial infarction.

**Case presentation:**

We report the case of a 58-year-old female patient complaining of new onset thoracic pain and shortness of breath. Electrocardiographic examination results were suggestive of an acute inferior myocardial infarction. However, echocardiography revealed significant pericardial tamponade. The cause was found to be a needle which remained inside the pelvis following a previous cesarean delivery, which the patient had undergone 18 years prior. In emergency setting, the needle was removed and the pericardial tamponade was resolved. Due to the prompt and efficient management, the patient had an uneventful postoperative recovery and presented no recurrence at the follow-up examinations.

**Conclusions:**

The migration of foreign bodies through tissues is exceedingly rare. If present, it may cause life-threatening complications. Since the aetiology of pericardial tamponade is vast, a thorough assessment is highly important. Therefore, echocardiography is the imaging modality of choice. We wish to highlight the possibility of migrating foreign bodies as probable cause for pericardial tamponade, as well as the importance of echocardiographic methods in the fast-track evaluation of such critical conditions.

## Background

Pericardial tamponade (PT) is often a life-threatening medical condition which prompts accurate and efficient diagnosis and treatment. The typical clinical picture of acute PT includes hypotension, jugular venous distension and diminished heart sounds, classically known as Beck’s triad [1], but other signs and symptoms may be also present, such as pulsus paradoxus, chest pain, palpitations, dyspnea, anxiety, dizziness or syncope. Significant pericardial collections may cause electrocardiographic changes: low voltage, electrical alternans, PQ segment depression, and sinus tachycardia. Regarding the aetiology of PT, a number of diseases can be mentioned: inflammation of the pericardium (viral or bacterial pericarditis, autoimmune pericarditis), neoplastic or lymphatic diseases, uremic syndrome. PT may develop after interventional cardiological procedures or open heart surgery, or as a complication of myocardial infarction [2]. Less frequently, PT may be the result of hemorrhage caused by penetrating trauma either to the heart wall or the pericardium, as well as to other intrapericardial vascular structures. Pericardial penetration may occur as a result of direct traumatic or iatrogenic injuries. Perforation caused by pericardial foreign bodies, however, is a strange and unexpected occurrence. Most foreign bodies enter the pericardium either through direct chest trauma or migrating after being ingested, a commonly encountered situation especially in children [3]. The migration of foreign bodies on considerable lengths and through various tissues and natural barriers is very rare. We hereby describe the unusual case of a patient presenting with pericardial tamponade caused by a migrating needle which remained inside the pelvis following a cesarean delivery 18 years prior and resembling ST segment elevation myocardial infarction (STEMI).

## Case presentation

### Patient information

A 58-year-old female patient presented to her general practitioner’s (GP) office accusing progressive thoracic pain and shortness of breath. The symptoms had debuted a few days prior to the consultation. Her past medical history revealed gastroesophageal reflux disease with regular proton-pump inhibitor medicamentous treatment and a previous birth through cesarean delivery, which she had undergone 18 years prior in a foreign country.

**Fig. 1:**
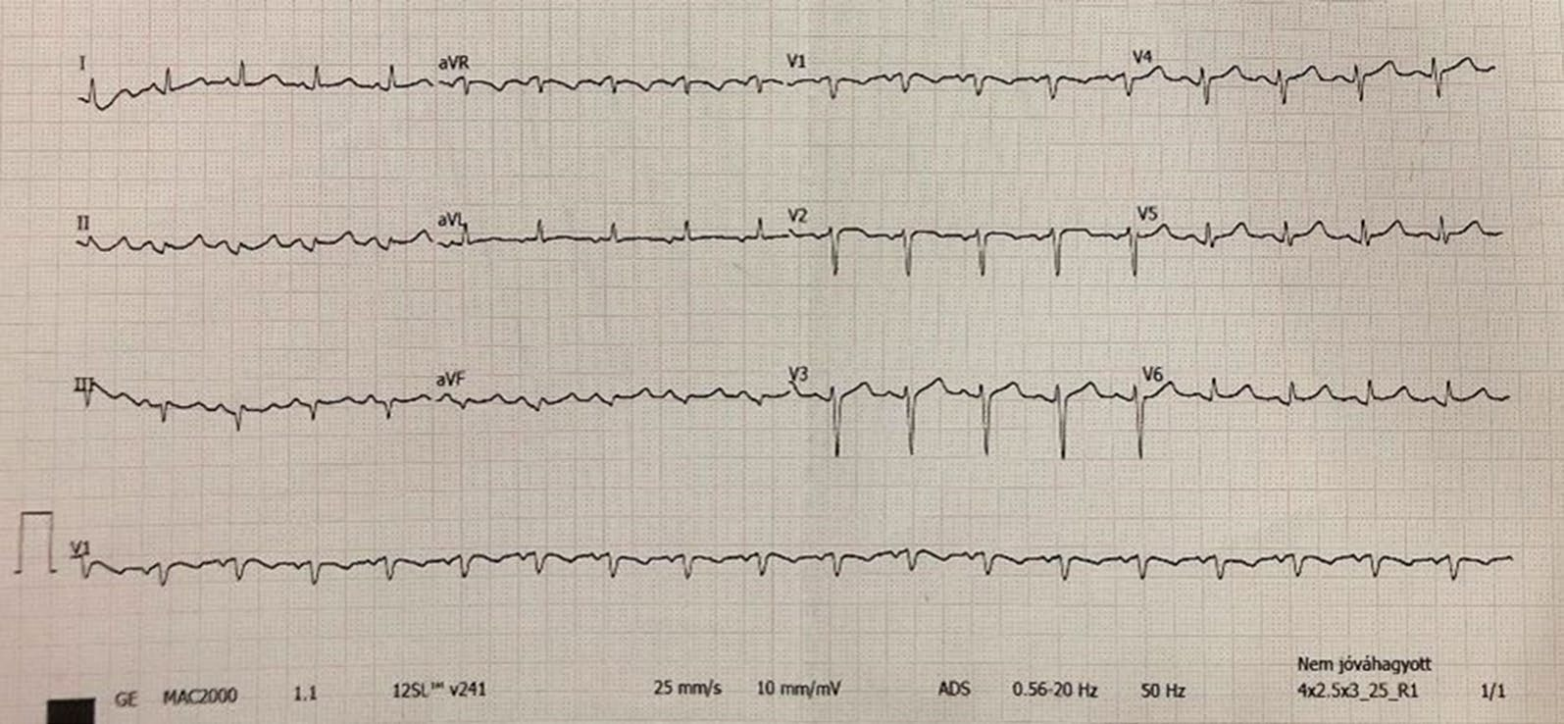
Electrocardiogram revealing sinus tachycardia (110 bpm), Q waves and minimal acute ST segment elevation with concordant T waves in the inferior leads

At the moment of consultation, her general status was mildly altered and she estimated the level of pain to be around 8/10. The physical examination conducted by the GP was marked by an episode of vomiting. On auscultation, all heart sounds were muffled. The patient’s blood pressure was 90/60 mmHg and her heart rate was 110 beats per minute. Electrocardiography (ECG) revealed sinus tachycardia, as well as Q waves and minimal acute ST segment elevation with concordant T waves in the inferior leads (Fig. 1). Due to the electrocardiographic aspect being suggestive of an acute inferior myocardial infarction (MI), the patient was referred to our clinic for further investigations and possible therapeutic intervention.

### Clinical findings

**Fig. 2:**
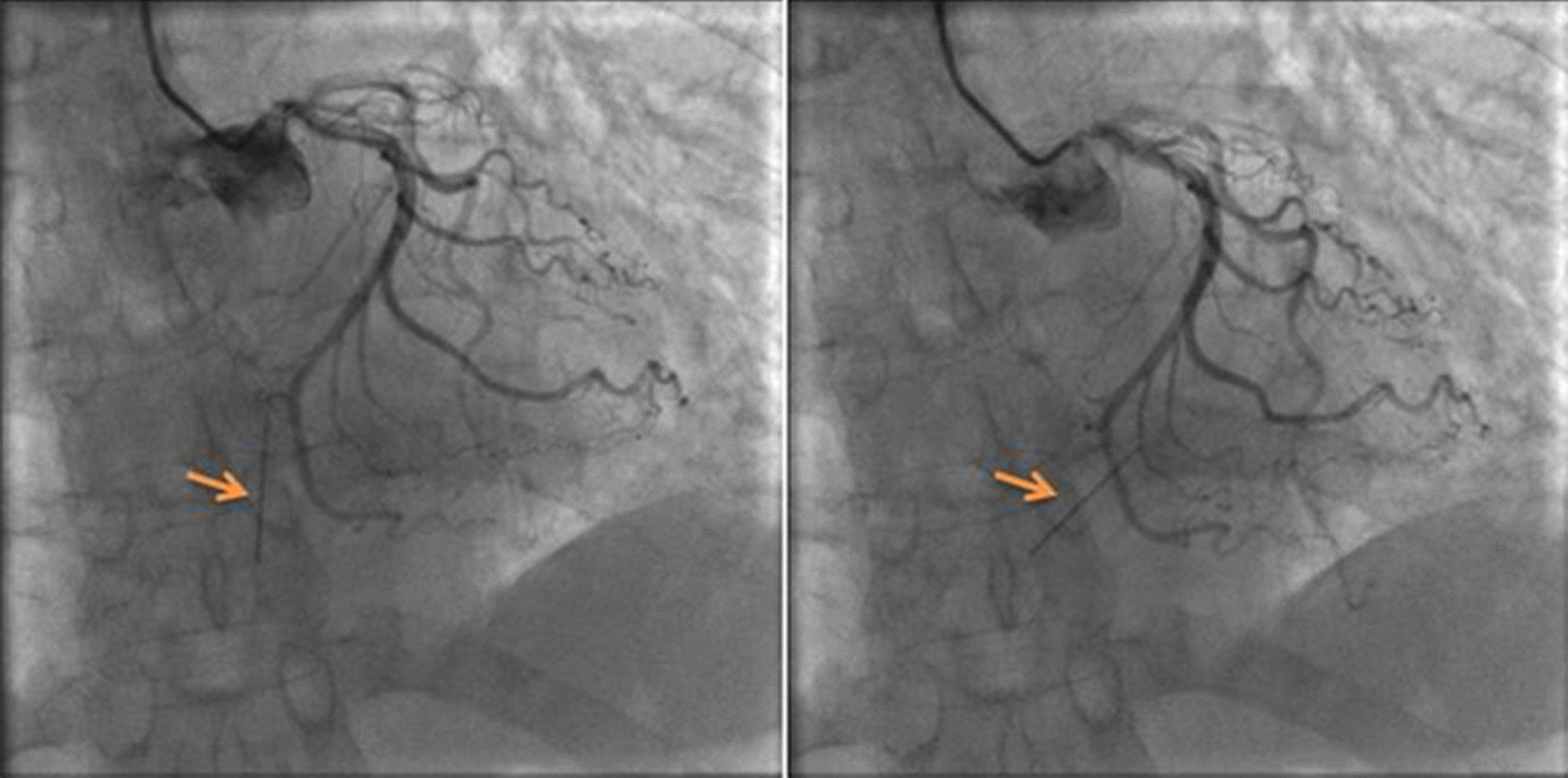
Cardiac catheterization: The silhouette of a needle-like object can be identified (orange arrows). It appears to be implanted into the heart, due to its rhythmic movements with the cardiac contractions

During the prehospital care the patient received 300 mg acetylsalicylic acid per os, 600 mg clopidogrel per os, and 5000 IU unfractionated heparin intravenously, in accordance to current STEMI guidelines [4, 5]. After admission, coronary angiography was promptly performed, but returned a negative result. However, it uncovered the silhouette of a long and thin metallic object (supposedly a needle) appearing to oscillate with the cardiac contractions (Fig. 2). After further inquiry, it was discovered that after the patient’s cesarean delivery 18 years prior, two needles remained inside her pelvis. They were initially kept under observation for a period of time, until one of them was removed after perforating through the abdominal wall.

### Diagnostic assessment and timeline

**Fig. 3:**
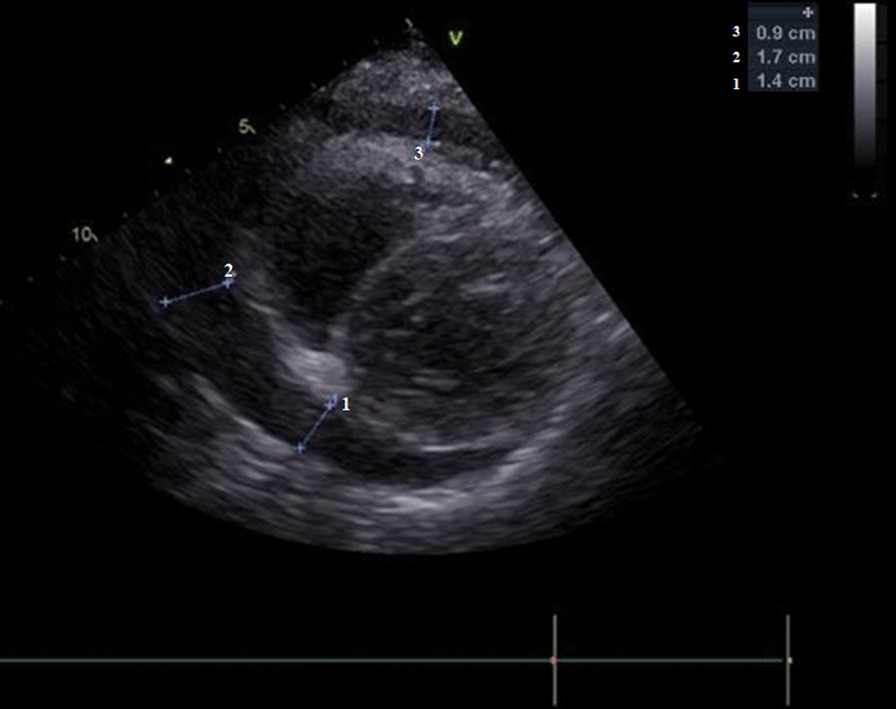
Echocardiographic imaging revealing the presence of significant pericardial collection

The patient was subsequently transferred to the cardiac intensive care unit (ICU), where she underwent bedside transthoracic echocardiography (TTE), which showed large pericardial effusion (Fig. 3). Arterial blood gas analysis and laboratory test results were within normal range, with a high-sensitivity cardiac troponin T (hs-cTnT) level of 9 ng/l. The sustained accumulation of the intrapericardial fluid, the slowly increasing heart rate with pulsus paradoxus and the persistent hypotension, along with the progression of dyspnoea at rest sustained the diagnosis of cardiac tamponade. Considering the clinical findings and the apparent cause of PT, the patient was scheduled to undergo emergency open-heart surgery.

### Therapeutic intervention

**Fig. 4:**
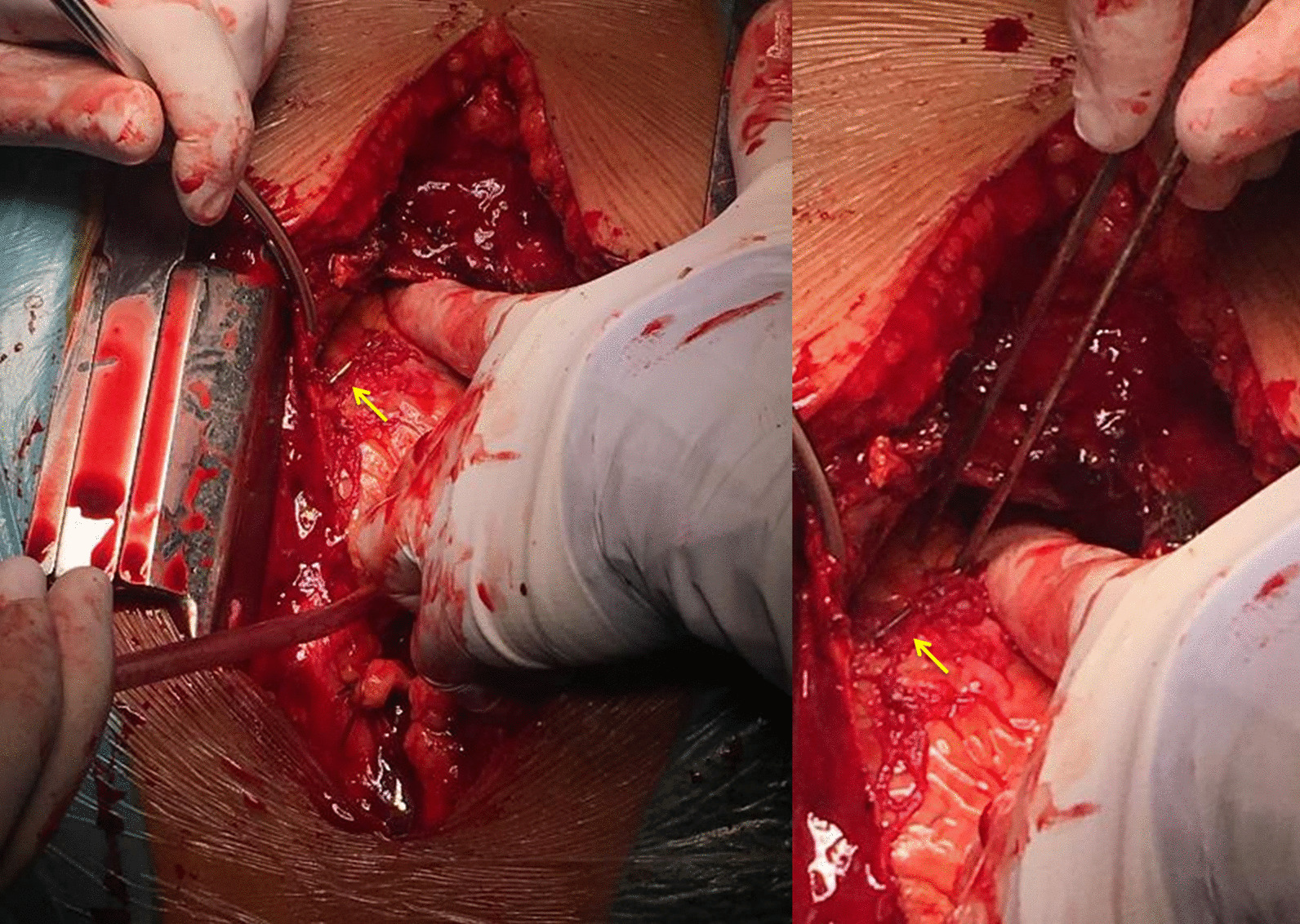
Intraoperative aspect after gaining access inside the pericardial space: the needle can be seen on the anterior surface of the heart (yellow arrow), being implanted into the cardiac wall and puncturing an epicardial artery, thus causing the intrapericardial bleeding

**Fig. 5:**
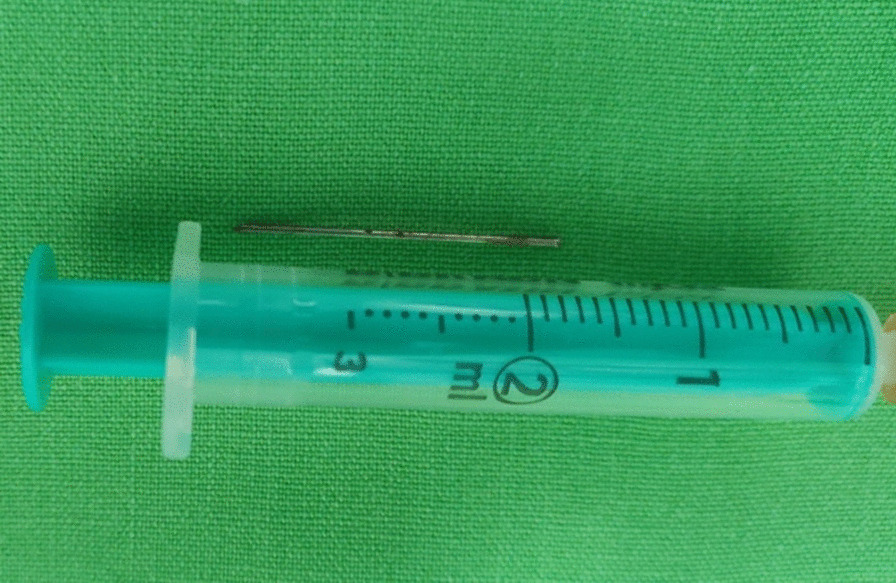
The needle after its surgical removal. The 3 ml syringe was used for size comparison

Following preoperative preparation, the patient was taken into the operating room. Upon gaining access inside the pericardial cavity, 300 ml of fresh arterial blood were evacuated, ergo promptly improving the patient’s haemodynamic condition. The foreign body was found to be penetrating the anterior wall of the right ventricle (Fig. 4). The source of the bleeding was identified to be an epicardial artery which was punctured by the needle. After the careful removal of the needle (Fig. 5), the bleeding was controlled by suturing the puncture site using a 5–0 polypropylene suture. The otherwise uneventful intervention was completed successfully after 70 min and the patient was transferred to the ICU.

### Follow-up and outcomes

Postoperatively, two units of red blood cells and eight units of platelet transfusion were administered. The patient was given analgesics, as well as ulcer prophylaxis. No pharmacological circulatory support was required. Extubation occurred at 3 h postoperatively, and the patient left the ICU the following morning. The postoperative ECG revealed no pathological findings. After an unremarkable postoperative period, she was discharged to a rehabilitation institution on the 7th day.

At the 1-year follow-up, the patient’s status was unaltered and TTE showed no recurrence.

### Discussions

Pericardial tamponade is a serious condition which may cause a wide spectrum of haemodynamic abnormalities, depending on its evolution (sudden or gradual). Most often, the rate with which the intrapericardial collection accumulates is more significant than its volume [6]. The accumulation of fluid or other contents inside the pericardial space may develop slowly in time, as it is the case of inflammatory or autoimmune diseases, infections or neoplasms, situations in which it may be well tolerated. However, a rapid increase in volume usually results in decreased cardiac output and severe circulatory distress [7] and it is commonly seen in pericardial or heart wall injuries caused by iatrogenic maneuvers, ventricular wall rupture (post MI) or other penetrating or blunt lesions. A traumatic injury to the heart wall caused by a migrated foreign body is, however, an uncommon situation. Still, it should not be overlooked in patients with history of invasive interventions.

The timely diagnosis of any pericardial effusion is critical, as it may rapidly evolve towards circulatory disturbances and even cardiogenic shock. A reliable and widely available imaging technique is bedside echocardiography, which allows the quick non-invasive evaluation of pericardial collections even in emergency situations, aiding in the efficient assessment of the patient’s status and providing therapeutic guidance [8]. In our case, due to fast-track examinations and prompt intervention, the patient’s status had not reached low cardiac output syndrome and the haemodynamic changes were well tolerated throughout the acute period, ultimately allowing the optimal postoperative recovery.

Considering that thoracic pain is a common manifestation in PT, the differential diagnosis should include all other acute disorders manifesting with chest pain, such as MI, pulmonary embolism or aortic dissection, but tension pneumothorax, peptic ulcer disease, gastritis or esophagitis should also not be omitted. The differential diagnosis should be based on other diagnostic methods as well, namely ECG and echocardiography, as well as chest radiography or computed tomography scans. As shown in our case report, PT can clinically resemble a MI. Therefore, ECG alone is not sensitive enough for a PT diagnosis. Echocardiography allows easy recognition of the presence and severity of PT, being the gold standard examination.

Foreign bodies are a more frequent occurrence in the spheres of gastroenterology and otolaryngology. Cases of ingested foreign bodies causing esophageal or gastric perforation and leading to PT, such as sewing needles, fish bones, or even pens, have been previously reported, in both children and adults [9–12]. Similarly, accidental or iatrogenic foreign body injuries to the heart have also been described as a possible cause of PT, among the incriminated objects being projectile fragments, catheter fragments, Kirschner wires, or acupuncture needles [13–16]. However, when aiming to identify the cause of intrapericardial bleeding in a patient with a history of previous surgical interventions, the possibility of a migrating foreign body should be taken into account. In uncommon cases, foreign bodies have been reported to migrate through tissues [17]. A possible explanation of this phenomenon is that suppuration causes the foreign bodies to dislodge and change position. This same mechanism also explains how such foreign bodies are sometimes eliminated through the skin or inside various cavities [18]. The loss of tissue elasticity which occurs in elderly patients as a result of loss of collagen may also be a predisposing factor [19]. Still, reports of foreign bodies that have migrated over long distances through the human body (e.g. pacemakers), are scarce [20]. Moreover, in contrast to the case we presented, the migration appears to occur most frequently downwards through the human body, presumably as a result of gravity. We theorize that the needle traveled in the opposite direction due to its reduced mass, but more detailed studies on the subject need to be conducted.

## Conclusions

Migrating foreign bodies are a rarely occurring situation. An exact mechanism for this phenomenon has not yet been defined. To the best of our knowledge, this is the firstly reported case of cardiac injury caused by a wandering foreign body, namely a remaining needle following cesarean delivery, and causing pericardial tamponade which mimicked ST segment elevation myocardial infarction. Moreover, it presents a rare example of long-distance chronic migration of a foreign body. By describing this case, we wish to highlight the possibility to encounter common pathologies with uncommon aetiologies in the regular medical practice, and to emphasize the importance of taking extraordinary differential diagnoses into account.

## Data Availability

Not applicable.

## Abbreviations

ECG: Electrocardiography
GP: General practitioner
hs-cTnT: High-sensitivity cardiac troponin T
ICU: Intensive care unit
MI: Myocardial infarction
PT: Pericardial tamponade
STEMI: ST segment elevation myocardial infarction
TTE: Transthoracic echocardiography
